# Supplementary material for: T cell responses to SARS-CoV-2 spike cross-recognize Omicron
Source: Nature. 2022 Jan 31;603(7901):488–92. doi: 10.1038/s41586-022-04460-3 (PMC8930768; doi:10.1038/s41586-022-04460-3)
Supplement: Supplementary file 2 — Reporting Summary [file 41586_2022_4460_MOESM2_ESM.pdf]

nature portfolio

Corresponding author(s): Catherine Riou
Wendy A. Burgers

Last updated by author(s): Jan 9, 2022

Reporting Summary

Nature Portfolio wishes to improve the reproducibility of the work that we publish. This form provides structure for consistency and transparency in reporting. For further information on Nature Portfolio policies, see our Editorial Policies and the Editorial Policy Checklist.

Statistics

For all statistical analyses, confirm that the following items are present in the figure legend, table legend, main text, or Methods section.

n/a	Confirmed	
☐	☒	The exact sample size (n) for each experimental group/condition, given as a discrete number and unit of measurement
☐	☒	A statement on whether measurements were taken from distinct samples or whether the same sample was measured repeatedly
☐	☒	The statistical test(s) used AND whether they are one- or two-sided<br>*Only common tests should be described solely by name; describe more complex techniques in the Methods section.*
☒	☐	A description of all covariates tested
☐	☒	A description of any assumptions or corrections, such as tests of normality and adjustment for multiple comparisons
☐	☒	A full description of the statistical parameters including central tendency (e.g. means) or other basic estimates (e.g. regression coefficient) AND variation (e.g. standard deviation) or associated estimates of uncertainty (e.g. confidence intervals)
☐	☒	For null hypothesis testing, the test statistic (e.g. F, t, r) with confidence intervals, effect sizes, degrees of freedom and P value noted<br>*Give P values as exact values whenever suitable.*
☒	☐	For Bayesian analysis, information on the choice of priors and Markov chain Monte Carlo settings
☒	☐	For hierarchical and complex designs, identification of the appropriate level for tests and full reporting of outcomes
☒	☐	Estimates of effect sizes (e.g. Cohen's d, Pearson's r), indicating how they were calculated

Our web collection on statistics for biologists contains articles on many of the points above.

Software and code

Policy information about availability of computer code

Data collection	Clinical data were recorded by trained clinicians using RedCap (version 9.5.36)
Data analysis	Statistical analysis were performed using GraphPad Prism version 9.3 Flow. Cytometry data were analysed with FlowJo Software (10.8, FlowJo LLC, BD Life Sciences) and Pestle and Spice v6.1 (https://niaid.github.io/spice). HLA prediction was performed using TepiTool from IEDB Analysis Resource (http://tools.iedb.org).<br>The quality control checks on raw sequence data and the genome assembly were performed using Genome Detective 1.133 (https://www.genomedetective.com). Phylogenetic classification of the genomes was done using the PANGOLIN software suite (v1.2.106) (https://github.com/hCoV-2019/pangolin).

For manuscripts utilizing custom algorithms or software that are central to the research but not yet described in published literature, software must be made available to editors and reviewers. We strongly encourage code deposition in a community repository (e.g. GitHub). See the Nature Portfolio guidelines for submitting code & software for further information.

Data

Policy information about availability of data

All manuscripts must include a data availability statement. This statement should provide the following information, where applicable:

- Accession codes, unique identifiers, or web links for publicly available datasets
- A description of any restrictions on data availability
- For clinical datasets or third party data, please ensure that the statement adheres to our policy

Datasets (raw data) underlying the figures have been provided as Source Data. Complete genome sequences for the viral isolates were deposited in GISAID.

Field-specific reporting

Please select the one below that is the best fit for your research. If you are not sure, read the appropriate sections before making your selection.

☒ Life sciences ☐ Behavioural & social sciences ☐ Ecological, evolutionary & environmental sciences

For a reference copy of the document with all sections, see nature.com/documents/nr-reporting-summary-flat.pdf

Life sciences study design

All studies must disclose on these points even when the disclosure is negative.

Sample size	Sample size was based on available samples rather than on a pre-defined samples size calculation
Data exclusions	Sample (PBMC) with low viability (<60%) or low cell number (CD4+ T cells < 20,000 cells) were excluded from the analysis.
Replication	Samples for each patient were analysed once due to limited availability
Randomization	As this is a observational study, randomization is not applicable.
Blinding	For flow cytometry assay, samples were stained and acquired in 7 consecutive runs over 2 weeks. While performing the experiments, investigators were blinded to patient groups.

Reporting for specific materials, systems and methods

We require information from authors about some types of materials, experimental systems and methods used in many studies. Here, indicate whether each material, system or method listed is relevant to your study. If you are not sure if a list item applies to your research, read the appropriate section before selecting a response.

Materials & experimental systems

n/a	Involved in the study	
☐	☒	Antibodies
☐	☒	Eukaryotic cell lines
☒	☐	Palaeontology and archaeology
☒	☐	Animals and other organisms
☐	☒	Human research participants
☐	☒	Clinical data
☒	☐	Dual use research of concern

Methods

n/a	Involved in the study	
☒	☐	ChIP-seq
☐	☒	Flow cytometry
☒	☐	MRI-based neuroimaging

Antibodies

Antibodies used

Antibodies
purified NA/LE mouse anti-human CD28 (clone 28.2) BD Pharmingen Cat# 555725; RRID:AB_2130052, dilution 1/1000
purified NA/LE mouse anti-human CD49d (clone L25) BD Pharmingen Cat# 555501; RRID:AB_396068, dilution 1/1000
LIVE/DEAD™ Fixable VIVID Stain Invitrogen Cat# L34955, dilution 1/2500
CD14 Pac Blue (clone TuK4) Invitrogen Thermofisher Scientific Cat# MHCD1428; RRID:AB_10373537, dilution 1/100
CD19 Pac Blue (clone SJ25-C1) Invitrogen Thermofisher Scientific Cat# MHCD1928; RRID:AB_10373689, dilution 1/100
CD4 PERCP-Cy5.5 (clone L200) BD Biosciences Cat# 552838; RRID:AB_394488, dilution 1/100
CD8 BV510 (clone RPA-8) Biolegend Cat# 301048; RRID:AB_2561942, dilution 1/100
CD3 BV650 (clone OKT3) Biolegend Cat# 317324; RRID:AB_2563352, dilution 1/100
IFN-g Alexa 700 (clone B27) BD Biosciences Cat# 557995; RRID:AB_396977, dilution 1/250
TNF BV786 (clone Mab11) Biolegend Cat# 502948; RRID:AB_2565858, dilution 1/100
IL-2 APC (clone MQ1-17H12) Biolegend Cat# 500310; RRID:AB_315097, dilution 1/100

Validation

All antibodies used in this study are commercially available. All antibodies were validated by their manufacturers and were titered to define the optimal titer for positive and negative separation.

Eukaryotic cell lines

Policy information about cell lines

Cell line source(s)	HEK293T-ACE2 cells were a gift from Dr Michael Farzan, Scripps, USA; H1299-E3 cell line was derived from H1299 (CRL-5803). H1299 cells were a gift from M. Oren, Weizmann Institute of Science.
Authentication	Cell lines were not authenticated
Mycoplasma contamination	Cell lines were tested for mycoplasma contamination and were mycoplasma negative.
Commonly misidentified lines (See ICLAC register)	None

Human research participants

Policy information about studies involving human research participants

Population characteristics	This study includes participants vaccinated with Ad26.COV2.S or Pfizer BNT162b2, convalescent COVID-19 patients and hospitalized COVID-19 patients. Samples were selected based on PBMC availability.<br>Demographic characteristics are presented in Extended Data Table 1. Clinical characteristics for each participants included in the study are presented in Extended Data Table 3 and 4.
Recruitment	Study participants vaccinated with Ad26.COV2.S. and convalescent donors were included in a prospective cohort study conducted at Groote Schuur Hospital (Western Cape). Pfizer BNT162b2 vaccinees were recruited from KwaZulu-Natal. Hospitalized COVID-19 patients were recruited from Groote Schuur and Tshwane hospitals. All participants were older then 18 years old, and all participants gave written informed consent. One bias that may be present is the ethnic background, aince ethnic background differs substantially between the different South African provinces. This could have implications for the prevalence of HLA class I and II molecules between patients recruited from different provinces.
Ethics oversight	The study was approved by the University of Cape Town Human Research Ethics Committee (ref: HREC 190/2020, 207/2020 and 209/2020) and the University of the Witwatersrand Human Research Ethics Committee (Medical) (ref. M210429 and M210752), the Biomedical Research Ethics Committee at the University of KwaZulu–Natal (ref. BREC/00001275/2020) and the University of Pretoria Health Sciences Research Ethics Committee (ref. 247/2020).

Note that full information on the approval of the study protocol must also be provided in the manuscript.

Clinical data

Policy information about clinical studies

All manuscripts should comply with the ICMJE guidelines for publication of clinical research and a completed CONSORT checklist must be included with all submissions.

Clinical trial registration	*Provide the trial registration number from ClinicalTrials.gov or an equivalent agency.*
Study protocol	*Note where the full trial protocol can be accessed OR if not available, explain why.*
Data collection	*Describe the settings and locales of data collection, noting the time periods of recruitment and data collection.*
Outcomes	*Describe how you pre-defined primary and secondary outcome measures and how you assessed these measures.*

Flow Cytometry

Plots

Confirm that:

☒ The axis labels state the marker and fluorochrome used (e.g. CD4-FITC).

☒ The axis scales are clearly visible. Include numbers along axes only for bottom left plot of group (a 'group' is an analysis of identical markers).

☒ All plots are contour plots with outliers or pseudocolor plots.

☒ A numerical value for number of cells or percentage (with statistics) is provided.

Methodology

Sample preparation	Blood was collected in heparin tubes and processed within 4 hours of collection. Peripheral blood mononuclear cells (PBMC) were isolated by density gradient sedimentation using Ficoll-Paque (Amersham Biosciences, Little Chalfont, UK) as per the manufacturer's instructions and cryopreserved in freezing media consisting of heat-inactivated fetal bovine serum (FBS, Thermofisher Scientific) containing 10% DMSO and stored in liquid nitrogen until use.

Instrument	BD Fortessa
Software	Flowjo V10.8.1, Pestle and Spice V6.1
Cell population abundance	No cell sorting was performed
Gating strategy	SARS-Cov-2-specific T cells were identified via the following gating strategy: Viable lymphocytes were identified by successive gating in singlets (FSC-A/FSC-H), time gate, live CD3 (SSC-A/ DUMP channel- dead cells, CD14, CD19,). Then, from live CD3+ T cells, CD4+ and CD8+ T cells were gated in CD4/CD8 plots. Next, SARS-CoV-2 -specific T cells were gated by plotting IFN-g, IL-2 and TNF-a

☒ Tick this box to confirm that a figure exemplifying the gating strategy is provided in the Supplementary Information.
